# FEMALE AND MALE GENETIC EFFECTS ON OFFSPRING PATERNITY: ADDITIVE GENETIC (CO)VARIANCES IN FEMALE EXTRA-PAIR REPRODUCTION AND MALE PATERNITY SUCCESS IN SONG SPARROWS (*MELOSPIZA MELODIA*)

**DOI:** 10.1111/evo.12424

**Published:** 2014-05-22

**Authors:** Jane M Reid, Peter Arcese, Lukas F Keller, Sylvain Losdat

**Affiliations:** 1 Institute of Biological and Environmental Sciences, School of Biological Sciences, Zoology Building, University of AberdeenTillydrone Avenue, Aberdeen, AB24 2TZ, Scotland; 2 Department of Forest and Conservation Sciences, 2424 Main Mall, University of British ColumbiaVancouver, BC, V6T 1Z4, Canada; 3 Institute of Evolutionary Biology and Environmental Studies, University of ZurichWinterthurerstrasse 190, 8057, Zurich, Switzerland

**Keywords:** Associative genetic effects, assortative reproduction, fertilization success, multiple mating, polyandry, reproductive strategy

## Abstract

Ongoing evolution of polyandry, and consequent extra-pair reproduction in socially monogamous systems, is hypothesized to be facilitated by indirect selection stemming from cross-sex genetic covariances with components of male fitness. Specifically, polyandry is hypothesized to create positive genetic covariance with male paternity success due to inevitable assortative reproduction, driving ongoing coevolution. However, it remains unclear whether such covariances could or do emerge within complex polyandrous systems. First, we illustrate that genetic covariances between female extra-pair reproduction and male within-pair paternity success might be constrained in socially monogamous systems where female and male additive genetic effects can have opposing impacts on the paternity of jointly reared offspring. Second, we demonstrate nonzero additive genetic variance in female liability for extra-pair reproduction and male liability for within-pair paternity success, modeled as direct and associative genetic effects on offspring paternity, respectively, in free-living song sparrows (*Melospiza melodia*). The posterior mean additive genetic covariance between these liabilities was slightly positive, but the credible interval was wide and overlapped zero. Therefore, although substantial total additive genetic variance exists, the hypothesis that ongoing evolution of female extra-pair reproduction is facilitated by genetic covariance with male within-pair paternity success cannot yet be definitively supported or rejected either conceptually or empirically.

Ongoing evolution of reproductive strategies, and of associated phenotypic traits, is widely hypothesized to stem from indirect selection resulting from genetic covariances among traits and fitness components expressed in females versus males (i.e., from cross-sex genetic covariances; Kirkpatrick and Barton 1997; Mead and Arnold 2004; Brommer et al. 2007; Kruuk et al. 2008). Such evolutionary hypotheses are particularly insightful when they not only propose that the key cross-sex genetic covariances exist, but also explain how those covariances can themselves result from assortative reproduction among interacting females and males (thereby creating linkage or gametic phase disequilibria). For example, assortative reproduction between males expressing exaggerated secondary sexual traits and females expressing corresponding mating preferences is widely understood to create cross-sex genetic covariances between trait and preference that can drive further “runaway” coevolution (Kirkpatrick and Barton 1997; Mead and Arnold 2004; Kokko et al. 2006). Analogous cross-sex genetic covariances have also been hypothesized to arise between polyandry (defined as female mating with multiple males within a single reproductive episode) and traits that increase a male's success in resulting competition for paternity, thereby facilitating ongoing coevolution of polyandry and paternity success (Jennions and Petrie 2000; Pizzari and Birkhead 2002; Simmons 2005; Evans and Simmons 2008). However, it remains conceptually and empirically unclear whether such genetic covariances could or do emerge within naturally polyandrous reproductive systems.

Polyandry has profound consequences because it creates postcopulatory sexual selection and can alter the overall magnitude of sexual selection in both sexes (Pizzari and Birkhead 2002; Simmons 2005; Kvarnemo and Simmons 2013; Parker and Birkhead 2013). Yet, the evolution and persistence of polyandry remains puzzling, particularly in circumstances where female multiple mating seems likely to be costly and hence directly selected against (Keller and Reeve 1995; Kvarnemo and Simmons 2013; Parker and Birkhead 2013, but see Simmons 2005). One intriguing and influential hypothesis is that ongoing evolution of polyandry is facilitated by cross-sex genetic covariances that result from the male–male competition for paternity that polyandry itself intrinsically generates (Keller and Reeve 1995; Evans and Simmons 2008). Specifically, offspring of polyandrous females will, by definition, be predominantly sired by males that are relatively successful in competition for paternity. If there were additive genetic variance in both polyandry and competitive paternity success, then cross-sex genetic covariance might arise due to linkage disequilibria resulting from the inevitable assortative reproduction between polyandrous females and successful sires. Polyandry might then evolve through indirect selection, assuming that paternity success is positively genetically correlated with male fitness (Keller and Reeve 1995; Jennions and Petrie 2000; Pizzari and Birkhead 2002; Simmons 2005; Evans and Simmons 2008).

This conceptual framework explaining polyandry is commonly phrased in terms of sperm competition; males that are successful sperm competitors will sire more offspring of polyandrous females than males that are less successful sperm competitors, potentially causing cross-sex genetic covariance between polyandry and sperm competitiveness and consequent coevolution (Keller and Reeve 1995; Pizzari and Birkhead 2002; Evans and Simmons 2008). However, it is a male's overall paternity success defined as his probability of fertilizing an available ovum, not solely his sperm competitiveness per se, that might ultimately underpin indirect selection on polyandry (Keller and Reeve 1995; Yasui 1997; Parker and Birkhead 2013). Indirect selection could consequently stem from genetic covariances with other traits that increase male paternity success rather than solely sperm competitiveness, potentially including copulation frequency or mate guarding, and will therefore stem from genetic covariance between polyandry and paternity success itself (Keller and Reeve 1995; Kvarnemo and Simmons 2013). Any ongoing evolution of polyandry will then depend on the genetic covariances among multiple traits that contribute to overall paternity success and other components of male and female fitness (Yasui 1997; Evans and Simmons 2008; Bilde et al. 2009). These covariances might be positive or negative depending on the existence and magnitude of trade-offs among key traits within or between the sexes (Moore et al. 2004; Simmons and Moore 2009; Evans 2010; Droge-Young et al. 2012; Pischedda and Rice 2012; Parker and Birkhead 2013).

Such expectations become particularly complex in systems where there are multiple potentially conflicting routes to reproductive success, such as consort versus sneaker or satellite matings, within-pair versus extra-pair reproduction or divergent pre- and postcopulatory mate choice, that are employed by common or different subsets of males or females (Webster et al. 1995; Evans 2010; Fricke et al. 2010; Kvarnemo and Simmons 2013; Parker and Birkhead 2013). Synergistic or conflicting variation in male paternity success achieved through different routes could then arise, and be differentially associated with different female reproductive strategies. The net cross-sex genetic covariances that could arise due to assortative reproduction, and the resulting magnitude and direction of indirect selection on polyandry (or consequent extra-pair reproduction) then become much harder to conceptualize and predict.

Furthermore, cross-sex genetic covariances could also stem from pleiotropy rather than solely from assortative reproduction and consequent linkage disequilibria, for example, if particular alleles at specific loci directly influence both female and male mating rates (Halliday and Arnold 1987; Harano and Miyatake 2007; House et al. 2008; Forstmeier et al. 2011). Net genetic covariances that do not entirely match those expected given observed or expected assortative reproduction could then potentially arise. Given this complexity, empirical studies of genetic covariances between female reproductive strategy and male paternity success in natural polyandrous systems are required to test the broad hypothesis that female strategy is genetically correlated with male paternity success, and to consider the degree to which such covariances might arise as intrinsic outcomes of the reproductive system (Arnqvist and Kirkpatrick 2005; Evans and Simmons 2008; Simmons and Moore 2009).

One specific polyandrous reproductive strategy that still requires adequate evolutionary explanation is female extra-pair reproduction in socially monogamous systems, where many offspring are sired by males other than a female's socially paired mate (Jennions and Petrie 2000; Griffith et al. 2002; Arnqvist and Kirkpatrick 2005; Uller and Olsson 2008). Extra-pair reproduction is hard to explain because there might be negative direct selection on both sexes due to general ecological costs of the underlying multiple mating (e.g., energetic demands or disease or predation risk), on cuckolded males that rear unrelated offspring, and on females whose cuckolded mate reduces parental care (Jennions and Petrie 2000; Griffith et al. 2002; Westneat and Stewart 2003; Arnqvist and Kirkpatrick 2005; Parker and Birkhead 2013). Female extra-pair reproduction is therefore widely postulated to result from indirect selection stemming from cross-sex genetic covariances with components of male fitness (Halliday and Arnold 1987; Jennions and Petrie 2000; Arnqvist and Kirkpatrick 2005; Forstmeier et al. 2011).

Here, we first consider the degree to which an intrinsic additive genetic covariance between female liability for extra-pair reproduction and male liability for within-pair paternity success might be expected to result from inevitable assortative reproduction. These female and male liabilities both influence the paternity of jointly reared offspring and therefore shape a single phenotype of interest: the paternity status of offspring, which in turn defines the degree of extra-pair reproduction. We illustrate that the basic expectation of positive genetic covariance between polyandry and paternity success (e.g., Keller and Reeve 1995; Evans and Simmons 2008) becomes complicated when considering extra-pair reproduction rather than polyandry per se, and when there are multiple potentially conflicting routes to paternity. We then provide an empirical example by estimating additive genetic variances in female liability for extra-pair reproduction and male liability for within-pair paternity success, and the cross-sex genetic covariance between the two, in free-living song sparrows (*Melospiza melodia*), and hence consider the degree to which resulting extra-pair reproduction could continue to evolve due to selection on either or both sexes.

## Cross-Sex Genetic Covariance: Expectation

The basic hypothesis that polyandry (i.e., female multiple mating) will create positive genetic covariance between polyandry and male paternity success stems from the expected assortative reproduction between polyandrous females and males that are successful sires, not from assortative mating or pairing per se (Keller and Reeve 1995; Pizzari and Birkhead 2002; Evans and Simmons 2008). In the context of extra-pair reproduction, the degree to which a female will conceive offspring with her socially paired male will partly depend on the female's additive genetic liability for extra-pair reproduction (i.e., her liability to produce an extra-pair offspring (EPO) sired by an extra-pair male as opposed to a within-pair offspring (WPO) sired by her socially paired male) and the male's additive genetic liability for within-pair paternity success (i.e., his liability to sire an offspring produced by his socially paired female). Some form of assortative reproduction and hence intrinsic genetic covariance between these female and male liabilities might therefore be predicted, even given random social pairing. Furthermore, in most socially monogamous systems, most offspring are in fact sired by a female's socially paired male (Griffith et al. 2002; Sardell et al. 2010) and variance in within-pair paternity success can cause substantial variance in male fitness (Webster et al. 1995). One pertinent hypothesis is therefore that ongoing evolution and persistence of female extra-pair reproduction could be shaped by genetic covariance with male within-pair paternity success.

**Figure 1:**
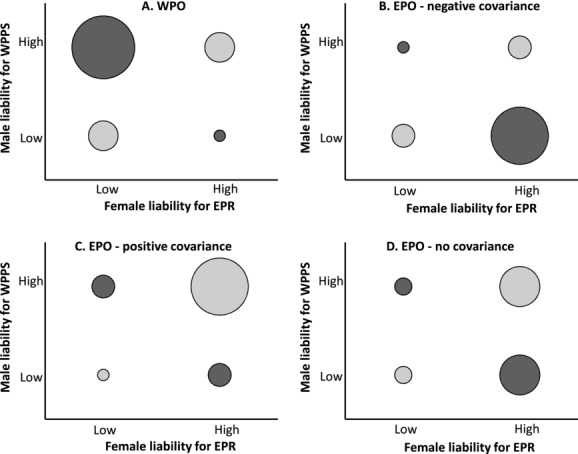
Conceptual view of the relative numbers of (A) within-pair offspring (WPO) and (B–D) extra-pair offspring (EPO) conceived by females and males with low or high additive genetic liabilities for extra-pair reproduction (EPR) and within-pair paternity success (WPPS), respectively, assuming (B) negative, (C) positive, or (D) zero genetic covariance between male within-pair paternity success and extra-pair reproductive success. Circle sizes indicate relative numbers of offspring. Dark and light shading indicates assortative reproduction that would, respectively, generate negative and positive genetic covariance between female liability for extra-pair reproduction and male liability for within-pair paternity success in offspring. These figures are intended to illustrate conceptual points not to be quantitatively accurate: absolute offspring numbers might differ across panels A versus B–D depending on the mean extra-pair reproduction rate, female and male genetic values will vary continuously rather than dichotomously, and offspring proportions will also depend on any assortative pairing or correlated variation in female fecundity.

However, the degree to which such genetic covariance could arise due to assortative reproduction might be constrained when females and males have both within-pair and extra-pair routes to reproduction. Assume, for initial simplicity, that social pairings form randomly with respect to female and male additive genetic liabilities for extra-pair reproduction and within-pair paternity success, respectively, and that female fecundity is independent of these values. Relatively many WPO will then be conceived by females with low genetic value for extra-pair reproduction and males with high genetic value for within-pair paternity success, creating negative genetic covariance across these WPO (Fig. 1A). In contrast, relatively few WPO will be conceived by females with high genetic value for extra-pair reproduction and males with low genetic value for within-pair paternity success, whereas intermediate numbers will be produced by females and males that both have low or high genetic values (Fig. 1A). The overall genetic covariance between female liability for extra-pair reproduction and male liability for within-pair paternity success that assortative reproduction generates across WPO might therefore be expected to be small, but unbalanced in that alleles underlying low female liability and high male liability will become associated more than alleles underlying the converse (Fig. 1A).

The total genetic covariance between female liability for extra-pair reproduction and male liability for within-pair paternity success will also depend on the covariance generated across EPO, many of which will by definition be conceived by females with high genetic value for extra-pair reproduction. This covariance will in turn depend on the genetic value for within-pair paternity success of males that sire EPO, and hence on the genetic covariance between male within-pair paternity success and extra-pair reproductive success. The latter covariance could be negative if there were a genetic trade-off between the two routes to male reproductive success, for example, if a male's ability to guard his socially paired female and hence defend his within-pair paternity impeded extra-pair mating and/or fertilization (Webster et al. 1995; Vedder et al. 2011). Indeed, negative genetic covariances between components of male mating and fertilization success have been predicted and observed (Moore et al. 2004; Evans 2010; Kvarnemo and Simmons 2013; Parker and Birkhead 2013). EPO would then tend to be sired by males with low genetic value for within-pair paternity success, creating some degree of negative genetic covariance between female liability for extra-pair reproduction and male liability for within-pair paternity success across EPO (Fig. 1B), complementing that generated across WPO (Fig. 1A).

Alternatively, the genetic covariance between male within-pair paternity success and extra-pair reproductive success could be positive, for example, if both traits were similarly affected by pleiotropic alleles influencing mating rate or sperm competitiveness. Most EPO might then be sired by males with high genetic value for within-pair paternity success, creating positive genetic covariance between female liability for extra-pair reproduction and male liability for within-pair paternity success across EPO (Fig. 1C). Relatively few EPO would, however, be conceived by females and males with low genetic values for extra-pair reproduction and within-pair paternity success, respectively (Fig. 1C). The overall genetic covariance between female liability for extra-pair reproduction and male liability for within-pair paternity success, conceptualized as a proportional combination of Figure 1A and C, might then be small because females with both high and low genetic values for extra-pair reproduction would conceive offspring with males with high genetic value for within-pair paternity success, creating opposing assortative reproduction through WPO and EPO. Additive genetic variance in female liability for extra-pair reproduction could then potentially be maintained by directional selection on male liability for within-pair paternity success. These scenarios imply that the overall reproductive system, including the form of the genetic covariance between male within-pair paternity success and extra-pair reproductive success, could then maintain evolutionary potential but constrain ongoing evolution of female extra-pair reproduction due to genetic covariance with male within-pair paternity success (rather than necessarily facilitate such evolution as suggested in the broader context of polyandry and paternity success, Keller and Reeve 1995; Evans and Simmons 2008).

However, the genetic covariance between male within-pair paternity success and extra-pair reproductive success might be close to zero, resulting in little genetic covariance between female liability for extra-pair reproduction and male liability for within-pair paternity success across EPO (Fig. 1D). The net covariance will also depend on any assortative social pairing or variation in female fecundity with respect to female and male genetic values for extra-pair reproduction and within-pair paternity success (which would alter the relative numbers of WPO and EPO produced by different parents), and on any direct pleiotropy. Empirical studies are therefore needed to quantify the realized additive genetic covariance between female liability for extra-pair reproduction and male liability for within-pair paternity success, and thereby to consider the degree to which female liability for extra-pair reproduction could potentially evolve through selection on male paternity success.

## Cross-Sex Genetic Covariance: Estimation

The hypothesis that female liability for extra-pair reproduction could evolve due to genetic covariance with male liability for within-pair paternity success requires that there is additive genetic variance in both liabilities and nonzero genetic covariance. The observed paternity of offspring produced by a female and reared with her socially paired male, and hence the observed degree of extra-pair reproduction, stems from the joint realization of these female and male liabilities. These liabilities can therefore be considered as direct and associative genetic effects of the female and her socially paired male on offspring paternity, meaning that the required genetic (co)variances can be estimated using quantitative genetic analysis of associative traits (e.g., Bijma et al. 2007; Brommer and Rattiste 2008; Simmons and Moore 2009; Bijma 2011). We used 20 years of paternity data from socially monogamous but genetically polygynandrous song sparrows (*M. melodia*) to estimate the additive genetic (co)variances in female liability for extra-pair reproduction and male liability for within-pair paternity success, and thereby consider whether female extra-pair reproduction could potentially evolve due to indirect selection stemming from genetic covariance with male within-pair paternity success (or vice versa).

### STUDY SYSTEM

Mandarte Island, BC, Canada (approximately 6 hectares), holds a resident and primarily socially monogamous song sparrow population which has been studied intensively since 1975 and recently numbered 10–50 breeding pairs (Smith et al. 2006; Sardell et al. 2010). Both sexes can breed from age 1 year and have median reproductive life spans of 2 years (interquartile range 1–4 years), and pairs can rear up to three broods of offspring per year (Smith et al. 2006; Lebigre et al. 2012). Females incubate clutches (typically three or four eggs), whereas both socially paired parents defend the breeding territory and provision hatched offspring (Smith et al. 2006). Both sexes can form new social pairs between years and sometimes between breeding attempts within single years given mortality or divorce of their previous mate.

Each year, all nests were located, clutch sizes were recorded, and all offspring surviving to 6 days posthatch were marked with unique combinations of metal and colored bands to allow subsequent identification (Smith et al. 2006). The occasional immigrants to Mandarte (1.1 year^−1^ on average, which is sufficient to prevent inbreeding from accumulating) were mist-netted and banded soon after arriving. All social pairings of adults and hence the social parents of all offspring were identified, as were all adult males that remained socially unpaired due to the typically male-biased adult sex ratio (Sardell et al. 2010; Lebigre et al. 2012; Reid et al. 2014).

During 1993–2012, 99.6% of banded offspring and adults were blood sampled and genotyped at 13 polymorphic microsatellite loci to allow assignment of genetic parents. Bayesian full probability models assigned genetic sires to 99.7% of sampled offspring with ≥95% individual-level confidence (Sardell et al. 2010). Overall, 28% of offspring were assigned as EPO (Sardell et al. 2010; Reid et al. 2014), compared to 24% in a nearby mainland song sparrow population (Hill et al. 2011). The probability of excluding a female's socially paired male as sire averaged 0.9998. All genetic mothers matched those identified by behavioral observations (Sardell et al. 2010).

The paternity data were used to quantify the numbers of WPO and EPO within each brood, thereby simultaneously measuring the female's realized degree of extra-pair reproduction and the realized within-pair paternity success of her socially paired male.

### QUANTITATIVE GENETIC APPROACH

One fundamental assumption of quantitative genetics, that any focal trait conforms to multivariate normality (Lynch and Walsh 1998), is violated by female extra-pair reproduction and male within-pair paternity success measured as the numbers of EPO versus WPO in each brood. We therefore considered the production of an EPO versus a WPO as a threshold trait and modeled underlying female and male liabilities for extra-pair reproduction and within-pair paternity success, respectively (e.g., Lynch and Walsh 1998; Bennewitz et al. 2007; Reid et al. 2011a).

We first fitted two separate univariate animal models to data describing the paternity status of offspring (WPO or EPO) in each brood to independently estimate additive genetic variance (*V*_A_) in female liability for extra-pair reproduction across females that produced each brood, and in male liability for within-pair paternity success across males that were socially paired to these females and hence reared each brood, and to check for potential biases. We then fitted a single univariate animal model that simultaneously estimated *V*_A_ in both female and male liabilities and the additive genetic covariance between the two, thereby treating the single observed phenotype of offspring paternity status as a joint trait of the female and her socially paired male with direct and associative effects, respectively (e.g., Bijma et al. 2007; McGlothlin and Brodie 2009; Wilson et al. 2009). These models are described in detail below.

### INDEPENDENT FEMALE AND MALE EFFECTS

The separate animal models for female and male liabilities assumed binomial responses, with the numbers of EPO and WPO banded in each brood as respective numerators and the total offspring banded in each brood as denominator in both cases. High liabilities therefore indicate high female and male propensities for extra-pair reproduction and within-pair paternity success, respectively, and therefore describe opposing effects on the paternity of jointly reared offspring.

Models included variance–covariance structures of additive genetic random effects derived from pairwise kinship (*k*) coefficients calculated from pedigree data, allowing estimation of *V*_A_ (Kruuk 2004). Random year and individual effects were fitted to estimate year and “permanent individual” variances, where the latter are assumed to comprise permanent environmental and nonadditive genetic variances (Kruuk 2004). Random female-year or male-year effects were also fitted to account for any correlation among multiple broods reared by individuals within years, thereby estimating “individual-year” variance.

Animal models also included linear regressions on individual coefficient of inbreeding (*f*), thereby estimating inbreeding depression in female and male liabilities and ensuring that estimates of *V*_A_ were not inflated (Reid and Keller 2010). Models of male liability also included a linear regression on male age because preliminary analyses suggested that within-pair paternity success increased with age. In practice, estimates of *V*_A_ were similar when these regressions and random year effects were excluded. Further effects that could influence the observed degree of extra-pair reproduction were not modeled because our current aim was to partition rather than explain total phenotypic variation.

Estimates of *V*_A_ can be inflated if there are unmodeled maternal or paternal effects that increase phenotypic resemblance among siblings, and moreover such parental effects can alter evolutionary trajectories (Simmons 2003; Kruuk 2004). However, >50% of song sparrow mothers and fathers had only one recruited daughter or son that contributed phenotypic data (Supporting Information). Estimates of *V*_A_ are consequently unlikely to be substantially inflated by additional phenotypic resemblance among siblings caused by common parental effects, and further analyses substantiated this expectation (Supporting Information).

The full dataset considered offspring that survived to genetic paternity assignment at about 6 days posthatch and excluded offspring that died earlier. Across broods where ≥1 offspring was genotyped, the egg to genotyping survival rate was high (88%). However, apparent *V*_A_ in female and male liabilities for extra-pair reproduction and within-pair paternity success could still conceivably reflect *V*_A_ in pregenotyping mortality of offspring sired by different males rather than *V*_A_ in paternity at conception, affecting interpretation and evolutionary inference (García-González 2008; Droge-Young et al. 2012). To investigate the magnitude of such effects, further animal models were fitted to restricted datasets comprising breeding attempts where all eggs survived to genotyping, and hence where genetic sires were assigned to all conceived offspring.

### DIRECT AND ASSOCIATIVE EFFECTS

The above models independently estimated *V*_A_ in female liability for extra-pair reproduction and male liability for within-pair paternity success from the same observed phenotype (the numbers of EPO vs. WPO in a brood). To relax the assumption of independent female and male effects, we fitted a univariate animal model that considered offspring paternity status as a single joint female–male trait that is influenced by direct genetic and environmental effects of a female, and by associative genetic and environmental effects of her socially paired male, thereby taking a “variance partitioning” approach to quantifying associative effects (e.g., Bijma et al. 2007; Brommer and Rattiste 2008; McGlothlin and Brodie 2009; Wilson et al. 2009; Bijma 2011). Full technical details of the model are provided as Supporting Information. In summary, the model considered a single binomial trait with the numbers of WPO and total offspring per brood as binomial numerator and denominator with each observed brood included once, and therefore partitioned variation in the probability that an offspring produced by a female and reared with her socially paired male would be a WPO versus an EPO. The model included variance–covariance matrices of additive genetic random effects for the female and her socially paired male, and simultaneously estimated *V*_A_ in female liability for within-pair reproduction and male within-pair paternity success and the additive genetic covariance between the two. This cross-sex genetic covariance is estimable across observations of broods produced by related females and males (as defined by the underlying relationship matrix) rather than directly across broods reared by observed socially paired females and males (see Supporting Information).

Since the model defined the numerator as the number of WPO, it quantified female liability for within-pair reproduction rather than extra-pair reproduction. This reversal does not affect estimated variance components, but does affect the sign of genetic covariances and regression slopes. Presented values were therefore multiplied by −1 to allow direct interpretation in the context of female extra-pair reproduction. Positive or negative genetic covariance would therefore indicate that high female liability for extra-pair reproduction was associated with high or low male liability for within-pair paternity success, respectively, across opposite-sex relatives.

The joint univariate model included linear regressions on female and male *f* and male age, and included random year and individual female and male effects. Random effects of individual social pairings, both within a single year and across multiple years, were also modeled to account for correlations among multiple broods reared by individual pairings and hence estimate “pair-year” and “pair” variances (Supporting Information). Because some males and females reared only one brood and/or only ever reared broods with each other (see Results), power to distinguish permanent individual and pair effects was relatively low (Supporting Information). However, our current aim was not specifically to estimate these effects, and they were fitted to ensure independence of residual errors across multiple broods reared by individual females, males, and pairs. In practice, these variance components were estimated to be small (see Results), and conclusions remained similar when they were excluded. Because socially monogamous individuals and pairings often reared EPO, there was power to distinguish female (direct) and male (associative) genetic effects across all observed relatives.

### ANALYSIS IMPLEMENTATION

All available genetic parentage data and ancestral social parentage data were used to compile a complete pedigree for the song sparrow population spanning 1975–2012 (Supporting Information; Reid et al. 2011b, 2014). Standard algorithms were used to compute *k* and *f* values. Kinship between immigrants and existing Mandarte-hatched natives, and hence *f* of offspring of immigrant-native pairings, was defined as zero relative to the pedigree baseline (Reid et al. 2011b). Phenotypic data from broods produced or reared by immigrant females or males were excluded because *f* is undefined for immigrants (as opposed to their offspring). Sample sizes therefore differed among analyses that considered offspring paternity status as a female or male trait or both, depending on which parent(s) were immigrants. The pedigree structure meant that there was nonzero detected *k* among all Mandarte-hatched females and males whose extra-pair reproduction or within-pair paternity success was observed but also substantial variation in *k*, providing power for quantitative genetic analyses (Table 1, Supporting Information).

**Table 1:** Statistics describing the distributions of pairwise coefficients of kinship (*k*) and individual coefficients of inbreeding (*f*) among the sets of *N*_fem_ female, *N*_male_ male, and *N*_tot_ total song sparrows that contributed phenotypic data to analyses of (A) female liability for extra-pair reproduction, (B) male liability for within-pair paternity success, and (C) both liabilities and their covariance, or were retained in the respective pruned pedigrees. Full distributions of *k* are provided as Supporting Information

	Individuals in the pruned pedigree			Individuals that contributed phenotypic data				
		Mean *k* ± 1SD	Median (range) of *k*		Mean *k* ± 1SD	Median (range) of *k*	Mean *f* ± 1SD	Median (range) of *f*
(A)	*N*_tot_: 531	0.056 ± 0.046	0.053 (0.000 – 0.471)	*N*_fem_: 254	0.074 ± 0.040	0.066 (0.005 – 0.409)	0.067 ± 0.050	0.064 (0.000 – 0.305)
(B)	*N*_tot_: 559	0.055 ± 0.045	0.052 (0.000 – 0.471)	*N*_male_: 273	0.072 ± 0.038	0.064 (0.004 – 0.412)	0.062 ± 0.049	0.058 (0.000 – 0.305)
(C)	*N*_tot_: 666	0.058 ± 0.044	0.055 (0.000 – 0.471)	*N*_tot_: 514	0.073 ± 0.039	0.065 (0.004 – 0.463)		
				*N*_fem_: 250			0.067 ± 0.050	0.062 (0.000 – 0.305)
				*N*_male_: 264			0.062 ± 0.050	0.058 (0.000 – 0.305)

Models were fitted using Bayesian methods implemented in MCMCglmm 2.17 in R version 2.15.2 (Hadfield 2010; R Development Core Team 2012), using logit link functions. Pedigrees were pruned to focal individuals and their assigned ancestors. Priors on fixed effects were normally distributed with mean zero and large variance (10^8^). Priors on variance components were inverse Wishart distributed, and posterior distributions were robust to reasonable variation in prior specification including parameter expansion.

Heritabilities of female and male liabilities for extra-pair reproduction and within-pair paternity success (conditioned on fitted fixed effects) were estimated from the separate models as *h*^2^ = *V*_A_/(*V*_Total_ + π^2^/3) given logistic variance proportional to π^2^/3, where *V*_Total_ is the sum of all estimated variance components (Nakagawa and Schielzeth 2010). For comparison, data-scale heritabilities, which are not independent of phenotypic means, were estimated as *h*^2^ = {*V*_A_·*X*^2^/(1 + μ)^2^}/{(*V*_Total_)·*X*^2^/((1 + μ)^2^ + *X*·(1 − *X*))}, where *X* = μ/(1 + μ) and μ is the observed trait mean (Nakagawa and Schielzeth 2010; Reid et al. 2011a).

When additive genetic (co)variances in female liability for extra-pair reproduction and male liability for within-pair paternity success were simultaneously estimated within a single univariate model, total additive genetic variance (*V*_ATotal_) in liability for extra-pair reproduction, which measures the population's total potential for an evolutionary response to selection (Bijma 2011), was calculated as *V*_ATotal_ = *V*_AFem_ + 2cov_(AFem,AMale)_ + *V*_AMale_, where *V*_AFem_, *V*_AMale_, and cov_(AFem,AMale)_ are the female (direct) and male (associative) additive genetic variances and their covariance, respectively (Bijma et al. 2007; Wilson et al. 2009; Bouwman et al. 2010). Furthermore, the total phenotypic variance also depends on cov_(AFem,AMale)_ when interacting individuals are related, and was calculated as 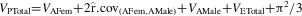 where 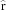 is the mean relatedness between socially paired females and males (Bouwman et al. 2010) and *V*_ETotal_ is the sum of all estimated nongenetic variance components. The ratio of *V*_ATotal_ to *V*_PTotal_ was calculated and compared to *h*^2^ for female liability for extra-pair reproduction, thereby allowing scale-free assessment of the contribution of associative genetic effects to the population's potential to respond to selection.

Analyses used 3,005,000 iterations, burn-in 5000 and thinning interval 3000, ensuring low autocorrelation among thinned samples (<0.05). Posterior means and 95% highest posterior density credible intervals (95% CI) for regression slopes, (co)variances, and heritabilities were estimated across thinned samples. Analyses of female liability for extra-pair reproduction differ from Reid et al. (2011a), which considered extra-pair reproduction per year rather than per brood. Raw means are presented ±1SD. Coefficients of additive genetic variance were not calculated because *V*_A_ was estimated on underlying liability scales. Data available from the Dryad Digital Repository: doi:10.5061/dryad.v7370.

## Results

### FEMALE LIABILITY FOR EXTRA-PAIR REPRODUCTION

During 1993–2012, Mandarte-hatched male song sparrows produced 966 broods where paternity was assigned to ≥1 offspring. The 966 broods were produced by 254 individual females (mean 3.8 ± 3.0 broods per female, range 1–16), of whom 54 (21%) contributed only one brood. The 966 broods were reared by 445 unique social pairings; individual females bred with a mean of 1.8 ± 1.1 (range 1–6) different socially paired males, although 148 (58%) females paired with only one male. A mean of 48.3 ± 18.5 broods produced by Mandarte-hatched females was observed per year (range 15–77), produced by 26.2 ± 10.7 individual females per year (range 9–43). Mean brood size across all 966 broods was 2.8 ± 1.0 banded offspring (median 3, range 1–4). Overall, 28.5% of offspring were assigned to an extra-pair sire, but proportional extra-pair reproduction within a brood varied from zero to one.

The animal model estimated substantial *V*_A_ in female liability for extra-pair reproduction (Table 2A). The posterior means for the permanent individual and individual-year variances were moderate, but the lower 95% CI limits converged toward zero (Table 2A). There was little among-year variance, but substantial residual variance (Table 2A). The posterior mean heritability of female liability for extra-pair reproduction was 0.22 (95% CI: 0.14–0.32, Table 2A). The regression on female *f* was negative, but the 95% CI substantially overlapped zero (Table 2A). Estimates of *V*_A_ and *h*^2^ were similar across 528 broods where paternity was assigned to all conceived offspring (Table 2B), and when maternal and paternal variances were also estimated (Supporting Information).

**Table 2:** Posterior mean variances, regression slopes, and heritabilities for (A and B) female liability for extra-pair reproduction (EPR) and (C and D) male liability for within-pair paternity success (WPPS) estimated from (A and C) all broods where at least one offspring survived to paternity assignment and (B and D) broods where paternity was assigned to the entire clutch, estimated from separate univariate animal models. Ninety-five percent credible intervals are in parentheses. *N*_obs_, *N*_fem_, and *N*_male_ are the numbers of broods, individual females, and individual males, respectively

			Variance components					Regressions		Heritabilities	
			Additive	Permanent		Individual-		Coefficient		Liability-	Data-
			genetic	individual	Year	year	Residual	of		scale	scale
	Model and data	Sample sizes	variance	variance	variance	variance	variance	inbreeding	Age	heritability	heritability
(A)	Female EPR.	*N*_obs_ = 966	2.23	0.25	0.04	0.88	3.28	−1.67	_____	0.22	0.18
	All broods.	*N*_fem_ = 254	(1.25–3.55)	(<0.001–0.81)	(<0.001–0.15)	(<0.001–1.72)	(2.13–4.39)	(−8.20–5.45)		(0.14–0.32)	(0.11–0.26)
(B)	Female EPR.	*N*_obs_ = 528	2.41	0.50	0.08	1.15	3.68	0.79	_____	0.21	0.18
	Entire clutch only.	*N*_fem_ = 210	(0.99–4.26)	(<0.001–1.58)	(<0.001–0.34)	(<0.001–2.73)	(1.92–5.67)	(-7.72–10.03)		(0.10–0.35)	(0.08–0.29)
(C)	Male WPPS.	*N*_obs_ = 998	1.07	0.27	0.08	1.20	3.41	1.76	0.24	0.11	0.10
	All broods.	*N*_male_ = 273	(0.21–1.98)	(<0.001–0.87)	(<0.001–0.31)	(0.001–2.17)	(2.09–4.68)	(-4.80–7.14)	(0.09–0.37)	(0.03–0.21)	(0.03–0.19)
(D)	Male WPPS.	*N*_obs_ = 553	1.07	0.21	0.09	1.22	4.19	0.09	0.33	0.11	0.10
	Entire clutch only.	*N*_male_ = 220	(0.14–2.14)	(<0.001–0.84)	(<0.001–0.31)	(<0.001–2.90)	(2.19–6.23)	(−7.33–6.65)	(0.17–0.51)	(0.02–0.21)	(0.02–0.19)

### MALE LIABILITY FOR WITHIN-PAIR PATERNITY SUCCESS

During 1993–2012, Mandarte-hatched male song sparrows reared 998 broods where paternity was assigned to ≥1 offspring. The 998 broods were reared by 273 individual males (mean 3.7 ± 3.0 broods per male, range 1–19), of whom 77 (28%) contributed only one brood. The 998 broods were reared by 457 unique social pairings; individual males bred with a mean of 1.7 ± 1.1 (range 1–7) different socially paired females, although 168 (62%) of males paired with only one female. A mean of 49.9 ± 19.2 broods reared by Mandarte-hatched males was observed per year (range 16–78), reared by 28.9 ± 12.1 individual males per year (range 12–48). Mean brood size across all 998 broods was 2.8 ± 1.0 banded offspring (median 3, range 1–4). Overall, 72.0% of offspring were assigned to the focal socially paired male, but proportional within-pair paternity success within a brood varied from zero to one.

The animal model estimated moderate *V*_A_ in male liability for within-pair paternity success (Table 2C). The posterior mean permanent individual variance was relatively small with a 95% CI that converged to zero, but there was nonzero individual-year variance (Table 2C). There was little among-year variance and substantial residual variance (Table 2C). The posterior mean heritability of male liability for within-pair paternity success was 0.11 (95% CI: 0.03–0.21, Table 2C). The regression on male age was positive, showing that older males had higher liability for within-pair paternity success (Table 2C). The posterior mean regression on male *f* was also positive, but the 95% CI overlapped zero (Table 2C). Estimates of *V*_A_ and *h*^2^ were similar across 553 broods where paternity was assigned to all conceived offspring (Table 2D), and when maternal and paternal variances were also estimated (Supporting Information).

### DIRECT AND ASSOCIATIVE EFFECTS

In total, 944 broods were reared by 434 different social pairings where both adults had hatched on Mandarte. These pairings involved 250 individual females and 264 individual males that respectively contributed means of 3.8 ± 2.9 (range 1–16) and 3.6 ± 3.0 (range 1–19) broods, but 52 (21%) females and 77 (29%) males contributed only one brood. These females and males socially paired with means of 1.7 ± 1.1 (range 1–6) and 1.6 ± 1.0 (range 1–7) different males and females, respectively. Although 147 (59%) and 166 (63%) females and males paired with only one social mate, only 58 (13%) pairings comprised females and males that only ever socially paired with each other. Mean *k* between a female and her paired social mate was 0.086 ± 0.054 (median 0.074, range 0.009–0.356), giving 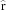 = 0.172.

The animal model that estimated direct effects of a female and associative effects of her socially paired male on the paternity of jointly reared offspring (and hence on the observed degree of extra-pair reproduction) estimated moderate *V*_A_ and *h*^2^ in both female liability for extra-pair reproduction and male liability for within-pair paternity success (Table 3). However, the posterior means were slightly smaller than those estimated from models that did not include additive genetic effects of the opposite sex fitted to the same broods (although the 95% CIs from each model included the posterior mean estimate from the other, Tables S1 and S2). The posterior mean additive genetic covariance was slightly positive, but the 95% CI was wide and overlapped zero (Table 3). The estimated year, permanent individual, pair, and pair-year variances were all relatively small (Table 3).

**Table 3:** Posterior mean (co)variances, regression slopes, and basic heritabilities for female liability for extra-pair reproduction and male liability for within-pair paternity success estimated from a univariate animal model that simultaneously considered both direct (female) and associative (male) effects on observed offspring paternity (and hence the observed degree of extra-pair reproduction). Ninety-five credible intervals are in parentheses. Data comprised 944 broods reared by 250 individual females and 264 individual males hatched within the study population. Estimates of the additive genetic covariance and female inbreeding depression are multiplied by −1 to allow direct interpretation in the context of female extra-pair reproduction rather than within-pair reproduction. Details of the univariate model structure are provided as Supporting Information

	Variance components							Regressions		Heritability
	Additive	Additive	Permanent		Pair-			Coefficient		Liability-
	genetic	genetic	individual	Pair	year	Year	Residual	of		scale
	variance	covariance	variance	variance	variance	variance	variance	inbreeding	Age	heritability
Female extra-pair	1.54	0.18	0.31	0.24	0.75	0.04	3.00	−1.80	_____	0.16
reproduction	(0.62–2.74)	(−0.38–0.87)	(<0.001–0.93)	(<0.001–0.80)	(0.001–1.66)	(<0.001–0.18)	(1.78–4.24)	(−8.04–4.63)		(0.07–0.26)
Male within-pair	0.48		0.15					1.69	0.26	0.05
paternity success	(0.11–1.01)		(<0.001–0.56)					(−4.32–7.25)	(0.09–0.39)	(0.01–0.11)

The total additive genetic variance in liability for extra-pair reproduction incorporating both direct and associative genetic effects was substantial (posterior mean *V*_ATotal_: 1.66, 95% CI: 0.56–3.30). The posterior mean total phenotypic variance (*V*_PTotal_) was 9.74 (95% CI: 8.11–11.84). The posterior mean ratio of *V*_ATotal_ to *V*_PTotal_ was therefore 0.17 (95% CI: 0.06–0.32), similar to the basic posterior mean *h*^2^ of female liability for extra-pair reproduction (Table 3).

## Discussion

Polyandry, and consequent extra-pair reproduction, is widespread in socially monogamous systems, but convincing demonstrations of evolutionary mechanisms operating in wild populations remain scarce (Arnqvist and Kirkpatrick 2005; Parker and Birkhead 2013). One overarching hypothesis is that polyandry and extra-pair reproduction could evolve due to positive cross-sex genetic covariances with components of male fitness (Halliday and Arnold 1987; Evans and Simmons 2008; Forstmeier et al. 2011). Most specifically, polyandry is hypothesized to create positive genetic covariance with male paternity success due to linkage disequilibria stemming from inevitable assortative reproduction (Keller and Reeve 1995; Pizzari and Birkhead 2002; Evans and Simmons 2008).

However, such hypotheses extrapolate from models that consider evolution of precopulatory mate choice (Keller and Reeve 1995; Jennions and Petrie 2000), or assume preexisting genetic covariances among life-history components (Yasui 1997), rather than explicitly considering what covariances could arise within complex reproductive systems. Genetic covariances, and corresponding evolutionary responses, could be constrained when there are multiple potentially conflicting routes to reproductive success in females and/or males, and further complicated when the trait of interest is extra-pair reproduction rather than polyandry per se (Moore et al. 2004; Evans and Simmons 2008; Evans 2010; Fricke et al. 2010). For example, the form of genetic covariance between female liability for extra-pair reproduction and male liability for within-pair paternity success stemming from assortative reproduction will depend on the genetic covariance between male liability for within-pair paternity success and extra-pair reproductive success (Fig. 1), as well as on any pleiotropy, assortative pairing, or correlated variation in fecundity. To understand ongoing evolution and persistence of extra-pair reproduction, one valuable empirical step is therefore to test the key hypotheses that there is nonzero additive genetic variance in female liability for extra-pair reproduction and male liability for within-pair paternity success, and estimate the cross-sex additive genetic covariance.

### GENETIC VARIANCES IN FEMALE AND MALE LIABILITIES

Analyses of song sparrow paternity data estimated nonzero *V*_A_ and *h*^2^ in both female liability for extra-pair reproduction and male liability for within-pair paternity success when both were treated as independent effects on offspring paternity status. Estimates remained quantitatively similar when analyses were restricted to broods where paternity was assigned to all conceived offspring. This suggests that estimates based on all broods, including those where some offspring died before paternity assignment, probably primarily reflect *V*_A_ in female and male liabilities for conceiving EPO versus WPO rather than solely *V*_A_ in postconception mortality of offspring sired by different males (e.g., García-González 2008; Droge-Young et al. 2012). These additive genetic variances imply that, all else being equal, there is potential for evolutionary responses to selection on offspring paternity status, and hence on the realized degree of extra-pair reproduction, both through females and through their socially paired males.

Explicit estimates of *V*_A_ in female propensity for polyandry or extra-pair reproduction (as opposed to repeat mating rate, e.g., Harano and Miyatake 2007; House et al. 2008) are scarce, especially in free-breeding populations (Simmons 2003; Evans and Simmons 2008; McFarlane et al. 2011; Reid et al. 2011a). Meanwhile, nonzero *V*_A_ in male fertilization success and associated traits has been widely estimated, albeit typically in highly constrained experimental or domesticated populations. For example, mean *h*^2^ for fertilization success was 0.15 across six species (Simmons and Moore 2009, see also Keller and Reeve 1995; Evans and Simmons 2008; Forstmeier et al. 2011). Such traits can also depend on maternal genetic and/or environmental effects, substantially altering expected evolutionary trajectories (Pizzari and Birkhead 2002; Simmons 2003; Evans and Simmons 2008; Simmons and Moore 2009). However, parental effects on female and male liabilities for extra-pair reproduction and within-pair paternity success were estimated to be relatively small in song sparrows (Supporting Information).

### GENETIC COVARIANCE

The existence of nonzero *V*_A_ in female and male liabilities for extra-pair reproduction and within-pair paternity success implies that there is potential for nonzero cross-sex genetic covariance affecting the realized paternity status of jointly reared offspring. This genetic covariance was estimated within a univariate animal model that simultaneously considered direct and associative genetic effects of a focal female and her paired social male on the single observed trait of offspring paternity status (i.e., WPO or EPO). Animal models estimate genetic (co)variances for basal populations, which in practice comprise pedigreed individuals with unknown parents. Unlike other forms of quantitative genetic analysis, estimates should consequently be unbiased by assortative reproduction among contemporary individuals (Lynch and Walsh 1998; Kruuk 2004). The estimated additive genetic covariance therefore pertains to basal individuals, not directly to the observed pattern of reproduction among contemporary females and males.

The posterior mean genetic covariance was slightly positive, where positive values would imply that females with high additive genetic liability for extra-pair reproduction have male relatives (not necessarily socially paired males in any observed instance) with high additive genetic liability to successfully defend the paternity of offspring produced by their own socially paired female. However, the 95% CI was wide and overlapped zero. These analyses therefore do not definitively support the hypothesis that ongoing evolution of female liability for extra-pair reproduction is facilitated by positive genetic covariance with male liability for within-pair paternity success, but do not definitively reject that hypothesis either.

Furthermore, the general expectation that positive genetic covariance between polyandry and paternity success will inevitably arise (Keller and Reeve 1995; Evans and Simmons 2008) does not necessarily hold in the context of extra-pair reproduction and within-pair paternity success (see *Cross-Sex Genetic Covariance: Expectation*). Some degree of positive genetic covariance could arise if a male's liability for within-pair paternity success were positively genetically correlated with his extra-pair reproductive success, creating diverging linkage disequilibria between female liability for extra-pair reproduction and male liability for within-pair paternity success across EPO compared to WPO (Fig. 1). Indeed, these within-pair and extra-pair components of male fitness are positively genetically correlated in song sparrows (additive genetic correlation 0.56, 95% CI: 0.01–0.81, Reid et al. unpubl. ms.). Female song sparrows with both low and high liabilities for extra-pair reproduction are therefore likely to conceive offspring with males with high liabilities for within-pair paternity success (Fig. 1A and C). The structure of the reproductive system, including the genetic covariance between male fitness components, may therefore constrain the overall genetic covariance between female liability for extra-pair reproduction and male liability for within-pair paternity success to be small (as estimated). This conclusion does not explain the origin of genetic variation in extra-pair reproduction, but may help explain why such substantial *V*_A_ in female liability for extra-pair reproduction remains.

Further explicit theory and empirical studies are required to consider whether analogous constraints arise in other systems where paternity depends on pairing status or mating order, creating positive or negative genetic covariances among paternity success achieved by individual males across different females (e.g., Pischedda and Rice 2012). In one study, Simmons (2003) estimated little *V*_A_, or hence genetic covariance, between measures of male fertilization success and female mating rate in field crickets (*Teleogryllus oceanicus*). In addition, cross-sex genetic covariances in repeat mating rate have been estimated to be small (e.g., Grant et al. 2005; Harano and Miyatake 2007) or positive (e.g., House et al. 2008) in invertebrate systems.

Precise estimation of cross-sex genetic covariances, particularly for liabilities underlying threshold traits, might require large breeding experiments that create numerous closely related males and females (e.g., House et al. 2008; Bilde et al. 2009; Evans 2010). However, such experiments might be inappropriate when the objectives are to estimate genetic covariances arising from natural reproductive systems (Kokko et al. 2006), or to estimate direct and associative genetic effects (Bijma et al. 2007). The song sparrow dataset, where extra-pair reproduction and within-pair paternity success were comprehensively observed across 20 years, is the most powerful such dataset currently available (Supporting Information). However, the 95% CI for the estimated genetic covariance was still wide. Further investigation and methodological development is required to determine whether this simply reflects low power, or whether it may also reflect unbalanced or nonlinear allelic associations and hence be biologically interesting.

### INDEPENDENT VERSUS ASSOCIATIVE GENETIC EFFECTS

Additive genetic variances in female and male liabilities for extra-pair reproduction and within-pair paternity success estimated from separate models that consider each effect on offspring paternity status independently should be unbiased if offspring reared by same sex relatives are randomly distributed across environments, including genetic environments posed by (unmodeled) opposite-sex relatives. This assumption underlies all quantitative genetic analyses where associative genetic effects are not explicitly considered. However, the univariate model that simultaneously estimated *V*_A_ in female and male liabilities returned smaller estimates than models that estimated *V*_A_ in each liability separately; posterior mean estimates of *V*_A_ and *h*^2^ for male liability for within-pair paternity success were halved. Estimates of *V*_A_ were therefore presumably biased when additive genetic effects of interacting individuals were not considered (see also Bijma et al. 2007; Bouwman et al. 2010). This may indicate some form of assortative pairing with respect to female and male liabilities for extra-pair reproduction and within-pair paternity success among related sparrows. Such patterns require future investigation, as does the degree to which offspring paternity also depends on the additive genetic liabilities for extra-pair paternity success of extra-pair males with whom socially paired females and males interact (e.g., Westneat and Stewart 2003; Simmons and Moore 2009). However, current analyses demonstrated substantial total additive genetic variance in extra-pair reproduction stemming from both direct (female) and associative (male) effects, suggesting that substantial combined evolutionary potential exists.

## DATA ARCHIVING

The doi for our data is 10.5061/dryad.v7370.

## Supporting Information

Disclaimer: Supplementary materials have been peer-reviewed but not copyedited.

Figure S1 Distributions of pairwise kinship (*k*) between (A) female and (B) male song sparrows whose extra-pair reproduction and within-pair paternity success were, respectively, observed during 1993–2012, and (C) between socially paired females and males. **Table S1**. Posterior mean variances, regression slopes and heritabilities for female liability for extra-pair reproduction estimated from (A) the basic univariate animal model fitted to all broods produced by Mandarte-hatched females during 1993–2012, and from this basic model: (B) plus random maternal effects fitted to all broods, (C) plus random social paternal effects fitted to all broods, (D) plus random maternal and social paternal effects fitted to all broods, (E) fitted to broods produced by a single daughter per mother, (F) fitted to broods produced by a single daughter per social father, (G) plus random maternal effects fitted to broods produced by females whose mothers contributed at least two daughters to the dataset, (H) plus random social paternal effects fitted to broods produced by females whose social fathers contributed at least two daughters to the dataset, and (I) fitted to broods produced and reared by females and males who had both hatched on Mandarte. **Table S2**. Posterior mean variances, regression slopes, and heritabilities for male liability for within-pair paternity success estimated from (A) the basic univariate animal model fitted to all broods reared by Mandarte-hatched males during 1993–2012, and this basic model: (B) plus random maternal effects fitted to all broods, (C) plus random social paternal effects fitted to all broods, (D) plus random maternal and social paternal effects fitted to all broods, (E) fitted to broods reared by a single son per mother, (F) fitted to broods reared by a single son per social father, (G) plus random maternal effects fitted to broods reared by males whose mothers contributed at least two sons to the dataset, (H) plus random social paternal effects fitted to broods reared by males whose social fathers contributed at least two sons to the dataset, and (I) fitted to broods produced and reared by females and males who had both hatched on Mandarte.
