# Supplementary material for: FEMALE AND MALE GENETIC EFFECTS ON OFFSPRING PATERNITY: ADDITIVE GENETIC (CO)VARIANCES IN FEMALE EXTRA-PAIR REPRODUCTION AND MALE PATERNITY SUCCESS IN SONG SPARROWS (MELOSPIZA MELODIA)
Source: Evolution. 2014 May 22;68(8):2357–70. doi: 10.1111/evo.12424 (PMC4285967; doi:10.1111/evo.12424)
Supplement: Figure S1 — Distributions of pairwise kinship (k) between (A) female and (B) male song sparrows whose extra-pair reproduction and within-pair paternity success were, respectively, observed during 1993–2012, and (C) between socially paired females and males. Table S1. Posterior mean variances, regression slopes and heritabilities for female liability for extra-pair reproduction estimated from (A) the basic univariate animal model fitted to all broods produced by Mandarte-hatched females during 1993–2012, and from this basic model: (B) plus random maternal effects fitted to all broods, (C) plus random social paternal effects fitted to all broods, (D) plus random maternal and social paternal effects fitted to all broods, (E) fitted to broods produced by a single daughter per mother, (F) fitted to broods produced by a single daughter per social father, (G) plus random maternal effects fitted to broods produced by females whose mothers contributed at least two daughters to the dataset, (H) plus random social paternal effects fitted to broods produced by females whose social fathers contributed at least two daughters to the dataset, and (I) fitted to broods produced and reared by females and males who had both hatched on Mandarte. Table S2. Posterior mean variances, regression slopes, and heritabilities for male liability for within-pair paternity success estimated from (A) the basic univariate animal model fitted to all broods reared by Mandarte-hatched males during 1993–2012, and this basic model: (B) plus random maternal effects fitted to all broods, (C) plus random social paternal effects fitted to all broods, (D) plus random maternal and social paternal effects fitted to all broods, (E) fitted to broods reared by a single son per mother, (F) fitted to broods reared by a single son per social father, (G) plus random maternal effects fitted to broods reared by males whose mothers contributed at least two sons to the dataset, (H) plus random social paternal effects fitted to broods [file evo0068-2357-sd1.pdf]

Supporting Information

FEMALE AND MALE GENETIC EFFECTS ON OFFSPRING PATERNITY: ADDITIVE GENETIC (CO)VARIANCES IN FEMALE EXTRA-PAIR REPRODUCTION AND MALE PATERNITY SUCCESS IN SONG SPARROWS (*MELOSPIZA MELODIA*)

Jane M. Reid, Peter Arcese, Lukas F. Keller and Sylvain Losdat

Maternal, paternal, individual and pair effects

The 254 female song sparrows that produced the 966 broods that contributed to basic estimation of additive genetic variance (V_A) in female liability for extra-pair reproduction were reared by 141 different mothers and 130 different social fathers, and originated from 187 different mother-years and 186 different social father-years and from 224 different broods. These 141 mothers and 130 social fathers had means of 1.8 ± 1.3SD (range 1-10) and 2.0 ± 1.4SD (range 1-7) daughters who contributed broods to the dataset respectively; 76 (54%) mothers and 68 (52%) social fathers had only one contributing daughter.

Similarly, the 273 male song sparrows that reared the 998 broods that contributed to basic estimation of V_A in male liability for within-pair paternity success were reared by 147 different mothers and 149 different social fathers, and originated from 213 different mother-years and 213 different social father-years and from 242 different broods. These mothers and social fathers had means of 1.9 ± 1.3SD (range 1-8) and 1.8 ± 1.2SD (range 1-6)

sons who contributed to the dataset respectively; 77 (52%) mothers and 82 (55%) social fathers had only one contributing son.

Estimates of V_A can be upwardly biased if there are common parental effects that increase phenotypic resemblance between offspring produced by individual mothers and/or fathers (i.e. between siblings, Kruuk 2004; Kruuk and Hadfield 2007). Bias due to 'permanent parental' effects (i.e. stemming from permanent environmental or non-additive genetic effects) can be minimised by modelling random maternal and paternal effects, and thereby accounting for phenotypic correlations among individuals that experienced similar parental environments and estimating maternal and paternal variances (V_{Mat} and V_{Pat}, Kruuk 2004; Kruuk and Hadfield 2007). Estimates of such parental variances are themselves of interest, because parental effects can substantially alter expected evolutionary trajectories.

The song sparrow dataset provides relatively low power to estimate V_{Mat} and V_{Pat} in female liability for extra-pair reproduction and male liability for within-pair paternity success because many mothers and social fathers had only one recruited daughter or one recruited son who contributed broods to the dataset (see above). There is consequently relatively high identity between 'permanent individual' effects and parental effects, meaning that the parental variances V_{Mat} and V_{Pat} will be hard to distinguish from permanent individual variance (V_{PI}). Furthermore, because some mothers and social fathers only produced recruited sons or daughters with one mate, V_{Mat} will be hard to distinguish from V_{Pat} (and from V_{Pair}, see below). However, the primary current objective was to obtain unbiased estimates of V_A in female liability for extra-pair reproduction and male liability for within-pair paternity success, not specifically to estimate V_{Mat}, V_{Pat} or V_{PI}. Power to distinguish V_{PI}

from residual variance (V_R) is also relatively low because ca.20% of daughters and sons only contributed one brood to the dataset. However permanent individual effects need to be modelled to ensure that estimates of V_A are not inflated by repeated (correlated) observations of individuals (Kruuk and Hadfield 2007).

Because relatively few song sparrow mothers or social fathers had multiple daughters or sons who contributed phenotypic data, any bias in estimates of V_A due to common maternal or paternal effects (and consequent increased phenotypic resemblance among siblings) is likely to be small even when these effects are not explicitly modelled (as in the main manuscript). However, to verify this expectation, we fitted three further sets of models:

First, we fitted random maternal and/or social paternal effects within the separate univariate animal models fitted to the full datasets for female liability for extra-pair reproduction and male liability for within-pair paternity success, thereby estimating V_{Mat} and/or V_{Pat}. Random effects of an individual's social father rather than genetic sire were fitted because all song sparrows are reared by their mother's socially paired male (even if they were sired by an extra-pair male). Any paternal environmental effects on offspring phenotype are therefore most likely attributable to the social father.

V_{Mat} and V_{Pat} were estimated to be relatively small (Table S1B and C, Table S2B and C). As expected the 95% CIs were wide, but the lower limit converged towards zero (Table S1B and C, Table S2B and C). Importantly, posterior mean estimates of V_A and h^2 were only slightly smaller than those estimated from models that did not include maternal or social paternal effects (Tables S1 and S2). Random effects of an individual female's or male's natal brood

were not fitted because there were few instances where multiple same-sex individuals recruited from the same brood (see above).

Second, we fitted basic animal models, without random parent effects, to datasets for female liability for extra-pair reproduction and male liability for within-pair paternity success that were restricted to broods produced by single daughters or sons per mother or social father. This restriction eliminated any potentially confounding non-independence of offspring within parents, and therefore eliminated any possibility for estimates of V_A to be inflated by unmodelled maternal or social paternal effects across siblings (while meaning that V_{Mat} and V_{Pat} cannot be distinguished from V_{PI}). To maximise statistical power, we retained broods produced by the daughter or son per mother or social father that produced most broods, selecting a single random daughter or son in cases where multiple offspring produced the same number of broods. Univariate animal models fitted to datasets produced by single realisations of this process returned estimates of V_A and h^2 that were broadly similar to those estimated from the full dataset, or if anything slightly higher (Table S1E and F, Table S2E and F).

Third, we fitted univariate animal models that included random maternal or social paternal effects to datasets for female liability for extra-pair reproduction and male liability for within-pair paternity success that were restricted to broods produced by daughters or sons whose mothers or social fathers produced at least two daughters or sons that contributed phenotypic data. This restriction maximised power to distinguish V_{Mat} and V_{Pat} from V_{PI}. Estimates of V_A and h^2 were very similar to those estimated by previous models (Table S1G

and H, Table S2G and H). V_{Mat} and V_{Pat} were estimated to be moderate, although 95%CIs still converged towards zero.

Estimates of V_A and h^2 from the different models presented within tables S1 and S2 are not strictly quantitatively comparable because models E-H are fitted to various different subsets of the full dataset used for models A-D. Furthermore, models E and F represent single realisations of a partially random data selection procedure, and there will also be some small Monte Carlo error. However, together, these additional analyses support the conclusion that there is non-zero V_A and h^2 in female liability for extra-pair reproduction and male liability for within-pair paternity success (even though power was reduced by restricting the dataset) when both are treated as independent traits, and that posterior mean estimates are not substantially confounded by maternal or social paternal environmental effects. These conclusions hold to the degree that it is feasible to distinguish V_A from V_{Mat} and V_{Pat} based on observational data (Kruuk and Hadfield 2007). Posterior mean estimates of inbreeding depression were less stable across the different models and datasets, but in all cases the 95%CIs substantially overlapped zero.

Similarly, in the univariate model that simultaneously estimated V_A and h^2 in female liability for extra-pair reproduction and male liability for within-pair paternity success as direct and associative genetic effects, permanent individual, pair and pair-year effects are to some degree confounded. This is because some males and females were only observed to breed with single social mates, or only produced one observed brood. However, our current aim was not to estimate the corresponding variances, and these effects were fitted to ensure

independence of residuals. In practice, estimates of V_A and h^2, and the genetic covariance, remained similar when pair and/or pair-year effects were excluded.

Table S1. Posterior mean variances, regression slopes and heritabilities for female liability for extra-pair reproduction estimated from A) the basic univariate animal model fitted to all broods produced by Mandarte-hatched females during 1993-2012, and from this basic model: B) plus random maternal effects fitted to all broods, C) plus random social paternal effects fitted to all broods, D) plus random maternal and social paternal effects fitted to all broods, E) fitted to broods produced by a single daughter per mother, F) fitted to broods produced by a single daughter per social father, G) plus random maternal effects fitted to broods produced by females whose mothers contributed at least two daughters to the dataset, H) plus random social paternal effects fitted to broods produced by females whose social fathers contributed at least two daughters to the dataset, and I) fitted to broods produced and reared by females and males who had both hatched on Mandarte. 95% credible intervals are in parentheses. N_{obs}, N_{fem}, N_{mother} and N_{father} are the numbers of broods, individual females, individual mothers and individual fathers respectively.

	Model and data	Sample sizes	Variance components							Regression	Heritabilities	
			Additive genetic variance (V_A)	Permanent individual variance (V_{PI})	Maternal variance (V_{Mat})	Paternal variance (V_{Pat})	Year variance (V_Y)	Individual-year variance (V_{IY})	Residual variance (V_R)	Coefficient of inbreeding (*f*)	Latent-scale heritability	Data-scale heritability
A)	Basic model. All broods.	N_{obs} = 966 N_{fem} = 254	2.23 (1.25-3.55)	0.25 (<0.001-0.81)	______	______	0.04 (<0.001-0.15)	0.88 (<0.001-1.72)	3.28 (2.13-4.39)	-1.67 (-8.20-5.45)	0.22 (0.14-0.32)	0.18 (0.11-0.26)
B)	Maternal variance. All broods.	N_{obs} = 966 N_{fem} = 254 N_{mother} = 141	1.99 (0.89-3.18)	0.19 (<0.001-0.67)	0.42 (<0.001-1.08)	______	0.04 (<0.001-0.14)	0.79 (<0.001-1.64)	3.31 (2.12-4.56)	-2.40 (-9.63-4.47)	0.20 (0.11-0.30)	0.16 (0.09-0.25)
C)	Paternal variance. All broods.	N_{obs} = 966 N_{fem} = 254 N_{father} = 130	2.01 (0.90-3.18)	0.21 (<0.001-0.71)	______	0.40 (<0.001-1.22)	0.04 (<0.001-0.15)	0.79 (<0.001-1.65)	3.31 (2.28-4.68)	-2.01 (-8.53-5.10)	0.20 (0.11-0.30)	0.16 (0.08-0.24)
D)	Maternal and paternal variances. All broods.	N_{obs} = 966 N_{fem} = 254 N_{mother} = 141 N_{father} = 130	1.83 (0.77-2.89)	0.17 (<0.001-0.61)	0.34 (<0.001-0.97)	0.35 (<0.001-1.09)	0.04 (<0.001-0.14)	0.78 (<0.001-1.63)	3.33 (2.15-4.67)	-2.75 (-9.78-4.40)	0.18 (0.09-0.29)	0.14 (0.07-0.23)
E)	Basic model. Single daughter per mother.	N_{obs} = 685 N_{fem} = 141	2.40 (1.05-3.98)	0.30 (<0.001-1.01)	______	______	0.06 (<0.001-0.26)	0.88 (<0.001-1.85)	3.06 (1.77-4.49)	5.32 (-2.31-13.60)	0.24 (0.12-0.36)	0.19 (0.09-0.29)
F)	Basic model. Single daughter per father.	N_{obs} = 656 N_{fem} = 130	2.77 (1.17-4.64)	0.32 (<0.001-1.04)	______	______	0.09 (<0.001-0.34)	0.76 (<0.001-1.74)	3.32 (2.00-4.78)	2.53 (-6.55-11.45)	0.26 (0.14-0.39)	0.21 (0.12-0.32)
G)	Maternal variance Multi-daughters per mother.	N_{obs} = 657 N_{fem} = 178 N_{mother} = 65	1.99 (0.80-3.44)	0.23 (<0.001-0.79)	0.43 (<0.001-1.29)	______	0.06 (<0.001-0.24)	0.64 (<0.001-1.53)	3.35 (1.99-4.78)	5.06 (-3.86-12.93)	0.20 (0.09-0.32)	0.16 (0.07-0.26)
H)	Paternal variance. Multi-daughters per father.	N_{obs} = 683 N_{fem} = 186 N_{father} = 62	2.00 (0.81-3.25)	0.15 (<0.001-0.60)	______	0.45 (<0.001-1.28)	0.05 (<0.001-0.21)	1.14 (<0.001-2.08)	2.74 (1.52-4.12)	4.62 (-2.20-12.90)	0.20 (0.10-0.32)	0.16 (0.08-0.26)
I)	Basic model. Both parents.	N_{obs} = 944 N_{fem} = 250	2.22 (1.08-3.45)	0.24 (<0.001-0.82)	______	______	0.03 (<0.001-0.12)	0.96 (0.001-1.78)	3.07 (1.84-4.27)	1.09 (-5.45-7.69)	0.22 (0.13-0.33)	0.21 (0.12-0.30)

Table S2. Posterior mean variances, regression slopes and heritabilities for male liability for within-pair paternity success estimated from A) the basic univariate animal model fitted to all broods reared by Mandarte-hatched males during 1993-2012, and this basic model: B) plus random maternal effects fitted to all broods, C) plus random social paternal effects fitted to all broods, D) plus random maternal and social paternal effects fitted to all broods, E) fitted to broods reared by a single son per mother, F) fitted to broods reared by a single son per social father, G) plus random maternal effects fitted to broods reared by males whose mothers contributed at least two sons to the dataset, H) plus random social paternal effects fitted to broods reared by males whose social fathers contributed at least two sons to the dataset, and I) fitted to broods produced and reared by females and males who had both hatched on Mandarte. 95% credible intervals are in parentheses. N_{obs}, N_{male}, N_{mother} and N_{father} are the numbers of broods, individual males, individual mothers and individual fathers respectively.

	Model and data	Sample sizes	Variance components							Regressions		Heritabilities	
			Additive genetic variance (V_A)	Permanent individual variance (V_{PI})	Maternal variance (V_{Mat})	Paternal variance (V_{Pat})	Individual-year variance (V_{IY})	Year variance (V_Y)	Residual variance (V_R)	Coefficient of inbreeding (*f*)	Age	Latent-scale heritability	Data-scale heritability
A)	Basic model. All broods.	N_{obs} = 998<br>N_{male} = 273	1.07 (0.21-1.98)	0.27 (<0.001-0.87)	_____	_____	1.20 (0.001-2.17)	0.08 (<0.001-0.31)	3.41 (2.09-4.68)	1.76 (-4.80-7.14)	0.24 (0.09-0.37)	0.11 (0.03-0.21)	0.10 (0.03-0.19)
B)	Maternal variance. All broods.	N_{obs} = 998<br>N_{male} = 273<br>N_{mother} = 147	0.95 (0.13-1.80)	0.24 (<0.001-0.81)	0.23 (<0.001-0.69)	_____	1.13 (<0.001-2.12)	0.08 (<0.001-0.30)	3.44 (2.18-4.59)	2.07 (-4.29-7.09)	0.24 (0.10-0.39)	0.10 (0.02-0.20)	0.09 (0.02-0.18)
C)	Paternal variance. All broods.	N_{obs} = 998<br>N_{male} = 273<br>N_{father} = 149	1.01 (0.13-1.87)	0.26 (<0.001-0.81)	_____	0.14 (<0.001-0.48)	1.18 (<0.001-2.17)	0.09 (<0.001-0.35)	3.44 (2.28-4.82)	2.09 (-3.00-7.94)	0.24 (0.10-0.37)	0.11 (0.02-0.20)	0.10 (0.02-0.18)
D)	Maternal and paternal variances. All broods.	N_{obs} = 998<br>N_{male} = 273<br>N_{mother} = 147<br>N_{father} = 149	0.94 (0.18-1.83)	0.23 (<0.001-0.79)	0.20 (<0.001-0.63)	0.11 (<0.001-0.42)	1.11 (0.001-2.07)	0.08 (<0.001-0.29)	3.51 (2.33-4.81)	2.40 (-3.31-8.75)	0.24 (0.09-0.38)	0.10 (0.02-0.19)	0.09 (0.02-0.17)
E)	Basic model. Single son per mother.	N_{obs} = 667<br>N_{male} = 147	0.68 (0.001-1.55)	0.31 (<0.001-1.07)	_____	_____	1.12 (0.001-2.12)	0.30 (<0.001-0.79)	2.86 (1.65-4.31)	5.05 (-1.41-10.79)	0.28 (0.13-0.42)	0.08 (0.001-0.19)	0.07 (0.001-0.18)
F)	Basic model. Single son per father.	N_{obs} = 677<br>N_{male} = 149	0.92 (0.001-1.83)	0.22 (<0.001-0.81)	_____	_____	1.64 (0.31-3.96)	0.14 (<0.001-0.46)	2.63 (1.38-3.96)	4.54 (-1.64-10.64)	0.27 (0.10-0.41)	0.15 (0.02-0.29)	0.13 (0.02-0.26)
G)	Maternal var. Multi-sons per mother.	N_{obs} = 728<br>N_{male} = 196<br>N_{mother} = 70	1.02 (0.001-2.25)	0.40 (<0.001-1.25)	0.39 (<0.001-1.10)	_____	1.55 (0.01-2.86)	0.11 (<0.001-0.43)	3.40 (1.90-4.80)	-0.63 (-8.85-7.26)	0.32 (0.15-0.49)	0.10 (0.01-0.24)	0.09 (0.01-0.22)
H)	Paternal var. Multi-sons per father.	N_{obs} = 689<br>N_{male} = 191<br>N_{father} = 67	1.25 (0.001-2.47)	0.44 (<0.001-1.42)	_____	0.22 (<0.001-0.73)	1.08 (<0.001-2.27)	0.15 (<0.001-0.57)	3.45 (1.87-4.94)	-0.63 (-8.98-6.57)	0.21 (-0.001-0.40)	0.12 (0.01-0.25)	0.12 (0.01-0.23)
I)	Basic model. Both parents.	N_{obs} = 944<br>N_{male} = 264	0.91 (0.01-1.78)	0.31 (<0.001-1.00)	_____	_____	1.66 (0.45-2.96)	0.07 (<0.001-0.27)	3.26 (1.98-4.56)	2.46 (-4.07-7.90)	0.25 (0.11-0.42)	0.10 (0.01-0.19)	0.09 (0.01-0.18)

Pedigree data and distributions of kinship coefficients

The genetic parentage data were used to compile a complete genetic pedigree for 1993-2012, with all banded sparrows assigned to their most probable genetic sire (Reid et al. 2014). Pedigree data derived from observed social parentage are available for 1975-1992 (Reid et al. 2014). Although these data presumably contain paternity error stemming from unknown extra-pair reproduction, all maternal links and ca.72% of paternal links will be correct assuming similar extra-pair paternity rates to those observed from 1993. They therefore contain useful information regarding relatedness among individuals that bred from 1993 onwards (and similar error to studies that assign genetic mothers and fathers with 80% statistical confidence). We therefore grafted the genetic pedigree for 1993-2012 onto the social pedigree for 1975-1992, thereby relaxing the alternative assumption that 1993 breeders were unrelated (Reid et al. 2011). To further minimise pedigree error, sparrows hatched during 1991-1992 that bred subsequently were genotyped to verify the paternity of individuals that contributed phenotypic data (Reid et al. 2014). Since the impact of ancestral pedigree error on estimated k among contemporary individuals decreases rapidly with increasing intervening generations, the error that remaining pre-1993 paternity error introduces into animal model estimates is most probably small (Reid et al. 2014, unpublished data).

Power to distinguish additive genetic variance (V_A) from other components of phenotypic variance depends on the kinship among individuals with observed phenotypes, and unbiased inference requires that relatives do not experience (unmodelled) common environmental effects. Moreover, estimating direct and associative genetic effects requires that groups of interacting individuals do not solely comprise close relatives (Bijma et al.

2007). In the current song sparrow dataset, there was some degree of detectable non-zero kinship among all females and males whose extra-pair reproduction or within-pair paternity success were observed and also substantial variation in kinship (Fig. S1), providing power to estimate V_A.

However, only 1% of pairwise *k* values among the 254 females that contributed phenotypic data exceeded 0.25, representing 208 mother-daughter comparisons and 106 full-sister comparisons (mostly involving sisters reared in different broods). A further 7% of pairwise *k* values exceeded 0.125, representing 149 grandmother-granddaughter comparisons and 2026 half-sister comparisons. Similarly, only 1% of pairwise *k* values among the 273 males that contributed phenotypic data exceeded 0.25, representing 233 father-son comparisons and 122 full-brother comparisons (mostly involving brothers reared in different broods). A further 6% of pairwise *k* values exceeded 0.125, representing 171 grandfather-grandson comparisons and 2080 half-brother comparisons. Most (92-93%) of comparisons among related females and males therefore involved distant relatives that are unlikely to share environmental, parental or dominance genetic effects (Fig. S1).

Of the 434 social pairings that contributed broods to the model that simultaneously considered female liability for extra-pair reproduction and male liability for within-pair paternity success, 13 (3%) were related by $k \geq 0.25$ (and were therefore full-sibling or parent-offspring pairs), and 60 (14%) were related by $k \geq 0.125$ (and were therefore half-sibling or grandparent-grandoffspring pairs, Fig. S1). Direct and associative genetic effects were therefore not completely confounded (e.g. Bijma et al. 2007).

Figure S1. Distributions of pairwise kinship (*k*) between (A) female and (B) male song sparrows whose extra-pair reproduction and within-pair paternity success were respectively observed during 1993-2012, and (C) between socially paired females and males. Descriptive statistics are provided in the main paper.

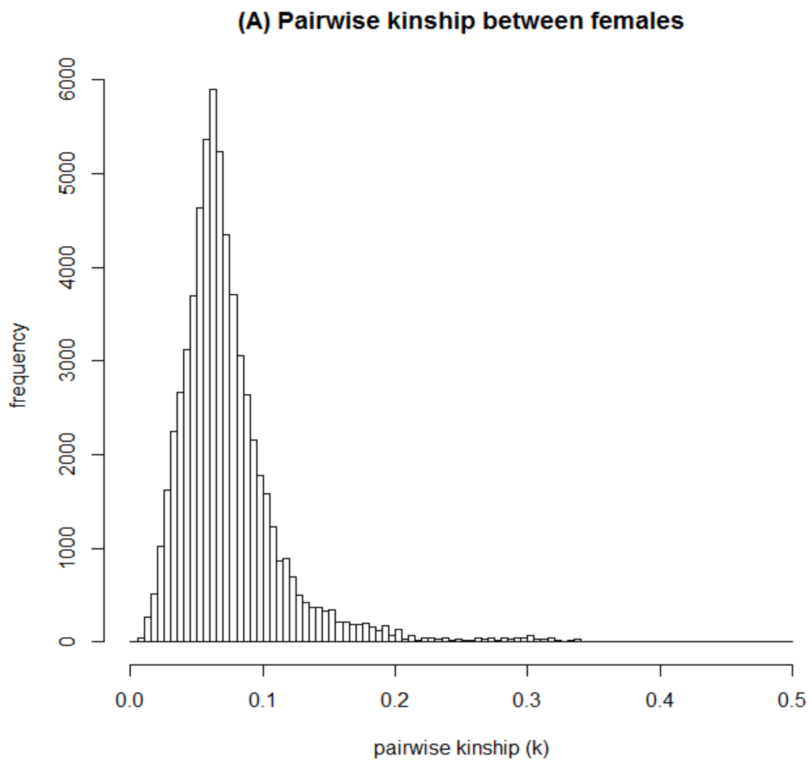

(B) Pairwise kinship between males

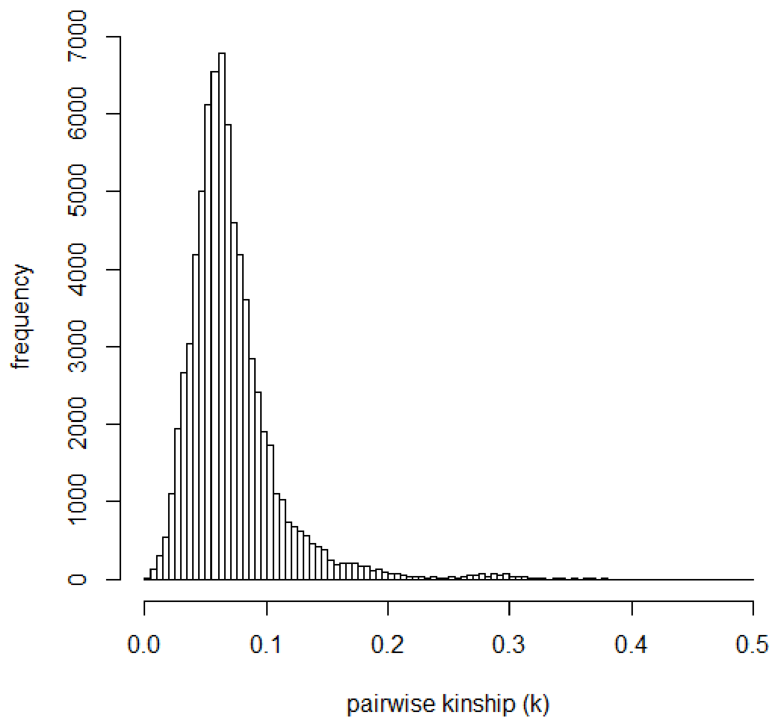

(C) Kinship between social pairings

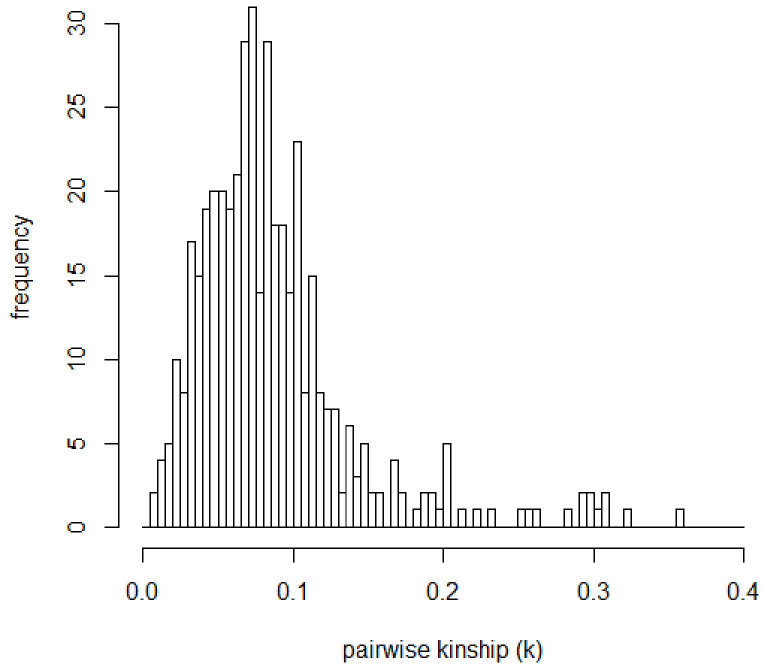

Associative genetic effects model

The univariate animal model used to simultaneously estimate additive genetic variance in female liability for extra-pair reproduction and male liability for within-pair paternity success (and hence direct (female) and associative (male) effects on the paternity of jointly reared offspring), and the additive genetic covariance between the two, was of the form:

$$\mathbf{l} = \mathbf{X\beta} + \mathbf{Z_{A.Fem}a_{Fem}} + \mathbf{Z_{A.Male}a_{Male}} + \mathbf{Z_{Fem.PI}PI_{Fem}} + \mathbf{Z_{Male.PI}PI_{Male}} + \mathbf{Z_{Pair}Pair} + \mathbf{Z_{PairYear}Pair.Year} + \mathbf{Z_{Year}Year} + \mathbf{e}$$

where **l** is the probability that an offspring will be a within-pair offspring (WPO) versus an extra-pair offspring (EPO) on the logit scale. The vector of observed phenotypes was assumed to be a (overdispersed) binomial variable with the numbers of WPO and total offspring observed within each brood as binomial numerator and denominator (giving a single observed phenotype per brood) and probability equal to the inverse logit of **l**; $\mathbf{a_{Fem}}$ and $\mathbf{a_{Male}}$ are vectors of additive genetic values for female and male liabilities respectively whose (co)variances are to be estimated; $\mathbf{PI_{Fem}}$ and $\mathbf{PI_{Male}}$ are vectors of random permanent individual effects for the female and her socially paired male respectively; **Pair**, **Pair.Year** and **Year** are vectors of random pair, pair-year and year effects; **β** denotes vectors of fixed effects (in the current case, male age and female and male coefficients of inbreeding); **X** and $\mathbf{Z_i}$ are appropriate design matrices respectively linking fixed and random effects to individuals; and **e** is a vector of residual errors giving a single residual for each observation in **l** (and accounting for overdispersion). This model therefore estimates female and male effects on the relative odds that a jointly reared offspring will be a WPO versus an EPO.

The covariance structure of the additive genetic effects is specified as a Kronecker product of the general form **A** $\otimes$ **G**. This product creates a block matrix containing all pairwise products of elements in **A** and **G**, where **A** is the matrix of additive genetic relationships between individuals (i.e. the numerator relationship matrix) and **G** is the additive genetic variance-covariance matrix for females and males. Because females and males can share genetic effects, the cross-sex genetic covariance for any observed trait is estimable from the probability that opposite-sex individuals share alleles identical by descent (as specified by the relationships described in **A**). Because the Kronecker product equates to the total variance-covariance matrix of additive genetic random effects, the estimated genetic covariance equates to the cross-sex genetic covariance between female and male liabilities (Lynch and Walsh 1998, p777; Bijma et al. 2007). More colloquially, this means that the cross-sex genetic covariance between female liability for extra-pair reproduction and male liability for within-pair paternity success can be estimated from brood compositions observed across female and male relatives (rather than directly across the socially paired female and male that reared each observed brood).

The model is univariate and includes a single phenotypic record per observed brood. It is important to conceptualise that it is not a bivariate model with two phenotypic records per observed brood considered as separate female and male traits. There is therefore no possible phenotypic covariance between female extra-pair reproduction and male within-pair paternity success within individual observed broods, because each brood is included only once. Furthermore, since female liability for extra-pair reproduction and male liability for within-pair paternity success are sex-limited traits, individuals cannot express both. Permanent individual effects on observed brood phenotypes originating from one sex therefore cannot be expressed through the other sex. The variances of $\mathbf{PI}_{\mathbf{Fem}}$ and $\mathbf{PI}_{\mathbf{Male}}$ are

therefore independent (and assumed identically distributed), and there can be no permanent individual covariance. The **Year**, **Pair** and **Pair.Year** effects account for any positive correlation across multiple broods reared in different years and by individual pairings across and within years, thereby ensuring independence of residuals across observed broods. Consequently, there is a single residual **e** for each individual observed brood, meaning that there is no identifiable residual covariance between female and male effects within broods. This does not imply that additional brood-specific random effects on female and male liabilities are not correlated within individual broods, but any such covariance cannot be estimated separately and is subsumed without bias within the total estimated residual variance.

This form of associative genetic effects model can be coded in R package MCMCglmm (Hadfield 2010) as:

model <- MCMCglmm(cbind(number.wpo, number.epo) ~ fixed.effects,

random = ~ str(female.animal + male.animal) + female.ID + male.ID + pair.ID + year,

family = family, data = data, prior = prior,

ginverse=list(female.animal=inv.A, male.animal=inv.A))

where inv.A is generated by:

inv.A <- inverseA(ped)$Ainv

and *number.wpo* and *number.epo* are the numbers of WPO and EPO observed in each brood which are then combined into a single binomial variable with *number.wpo* conditioned on brood size (*number.wpo* + *number.epo*).

LITERATURE CITED

Bijma, P., W. M. Muir, E. D. Ellen, J. B. Wolf, and J. A. M. van Arendonk. 2007. Multilevel selection 2: Estimating the genetic parameters determining inheritance and response to selection. Genetics 175:289-299.

Hadfield, J. D. 2010. MCMC methods for multi-response generalized linear mixed models: the MCMCglmm R package. J. Stat. Soft. 33:1-22.

Kruuk, L. E. B. 2004. Estimating genetic parameters in natural populations using the 'animal model'. Phil. Trans. R. Soc. B 359:873-890.

Kruuk, L. E. B., and J. D. Hadfield. 2007. How to separate genetic and environmental causes of similarity between relatives. J. Evol. Biol. 20:1890-1903.

Lynch, M., and B. Walsh. 1998. Genetics and analysis of quantitative traits. Sinauer, Sunderland.

Reid, J. M., P. Arcese, R. J. Sardell, and L. F. Keller. 2011. Additive genetic variance, heritability and inbreeding depression in male extra-pair reproductive success. Am. Nat. 177:177-187.

Reid, J. M., L. F. Keller, A. B. Marr, P. Nietlisbach, R. J. Sardell, and P. Arcese. 2014. Pedigree error due to extra-pair reproduction substantially biases estimates of inbreeding depression. Evolution, *online early*.
